# Morocco’s population contact matrices: A crowd dynamics-based approach using aggregated literature data

**DOI:** 10.1371/journal.pone.0296740

**Published:** 2024-03-14

**Authors:** Dramane Sam Idris Kanté, Aissam Jebrane, Adnane Boukamel, Abdelilah Hakim

**Affiliations:** 1 Complex Systems and Interactions Team, Ecole Centrale Casablanca, Bouskoura, Morocco; 2 LAMAI, Faculty of Sciences and Technology, Cadi Ayyad University, Marrakesh, Morocco; Qatar University, QATAR

## Abstract

Estimation of contact patterns is often based on questionnaires and time-use data. The results obtained using these methods have been used extensively over the years and recently to predict the spread of the COVID-19 pandemic. They have also been used to test the effectiveness of non-pharmaceutical measures such as social distance. The latter is integrated into epidemiological models by multiplying contact matrices by control functions. We present a novel method that allows the integration of social distancing and other scenarios such as panic. Our method is based on a modified social force model. The model is calibrated using data relating to the movements of individuals and their interactions such as desired walking velocities and interpersonal distances as well as demographic data. We used the framework to assess contact patterns in different social contexts in Morocco. The estimated matrices are extremely assortative and exhibit patterns similar to those observed in other studies including the POLYMOD project. Our findings suggest social distancing would reduce the numbers of contacts by 95%. Further, we estimated the effect of panic on contact patterns, which indicated an increase in the number of contacts of 11%. This approach could be an alternative to questionnaire-based methods in the study of non-pharmaceutical measures and other specific scenarios such as rush hours. It also provides a substitute for estimating children’s contact patterns which are typically assessed through parental proxy reporting in surveys.

## Introduction

Social contact matrices constitute a key tool for predicting the spread of close contact infections. The estimation of these matrices has been the subject of several studies and is still an active research topic. In prior studies [1–3], they proceeded to evaluate contact matrices using serological data. This approach requires some assumptions about the structure of the matrix. To design control strategies against infectious respiratory diseases, Edmunds et al. [4], introduced a method to directly quantify contact patterns using questionnaires where people noted down the age of each person they had a conversation with over the period of one, randomly assigned, day as well as the social context (home, workplace, etc.) where that conversation took place. A contact was defined as a two-way exchange at a distance that did not require raising the voice. At least two words must have been spoken by each party and no barrier should separate them. Researchers in [5] referred to self-reported data obtained during interviews where people were asked about information about people they conversed with. Recently two major studies aimed at estimating contact matrices stood out [6, 7]. However, these two methods use different methods. The POLYMOD project also relies on questionnaires to compute contact patterns. They define a contact as a two-way conversation of at least three words. The study was conducted in 8 European countries. In [8, 9], they projected the obtained matrices on 172 countries. These surveys rarely consider the temporal changes that shape the patterns of social contacts due to holidays, weekends, or seasonality. Recently, some studies addressed this limitation by studying the effects of seasonality on human-to-human interactions on social contact patterns [10, 11]. Moreover, collecting surveys requires a lot of time, effort and resources. Little Italy is an approach that reckons on agent-based models and numerical simulations to create “synthetic populations” to overcome that limitation. The model mimics a synthetic Italian society and was used to compute the rates of daily contacts in various locations [7]. Simulations can compute a random day in people’s weekly routines. They consider that a day starts at 4 am. Then, they divided it into 144 time slots of 10 minutes each. Individual displacement is not considered to be dynamic and is given by the questionnaire for each time slot. Contact is considered whenever two individuals share the same place. Another study [12] used the same definition of contact to quantify the contact patterns of 26 European countries. They relied on census data to generate virtual societies of agents and estimated contact patterns in different places of activity using ABM. Still, both approaches do not accurately capture the effect of rush hours, panic situations and physical distance on disease propagation [13].

Some researchers used the traditional method based on surveys to estimate contact patterns [14]. They estimated a reduction of 74% in the number of contacts which was consistent across age. Nevertheless, in [15], researchers suggest a high variability in contact patterns due to a proportion of the population still maintaining a high level of social interactions (health workers, providers of essential services). Another method consists in multiplying pre-pandemic contact matrices by time-dependent control functions that account for the start and the end of the control measure [16–18]. However, this approach also implies homogeneity in people’s social interactions when social distancing is adopted. After reviewing the currently used methods for determining contact matrices, we conclude that an efficient strategy for calculating these matrices should meet the following criteria: precision, ease of implementation, and consideration of social distancing. We propose a novel methodology that allows the integration of social distancing and other scenarios such as panic. Our method is based on a modified social force model. Social force models are a class of mathematical models that are used to simulate the movement of pedestrians in complex conditions such as crowded areas and panic situations. In these models, Newton’s second law of dynamics is used to compute the movement trajectories of pedestrians [19–21]. An important advantage of these models is that they are physics-based and thus can be analyzed to gain insights into contact patterns at specific locations. Social force models were also previously applied to study the transmission dynamics of infectious diseases. Indeed, a social force model was applied to quantify the impact of non-pharmaceutical interventions on indoor disease transmission [22]. We have previously used a social force model to develop a multi-scale framework that describes COVID-19 transmission dynamics [23–25]. Another significant feature of social force models is their ability to describe physical distancing. Indeed, these models assume that individuals tend to keep an interpersonal distance from others. The social force model (SFM) has been used to estimate contacts and exposure to infected individuals in different settings [26]. Researchers in [27] estimated contacts in an airplane boarding scenario to deduce the risk of exposure. In [28], they estimated contact durations in a supermarket. The SFM, coupled with an equation of pedestrian shopping behavior, allowed to estimate exposure times in a supermarket [29]. Researchers in [30] used an EXPOSED approach with the SFM to quantify contact durations in a railway station. Another study [31] considers that two individuals made a contact when they spent 15 minutes within 6 feet (1.83 m) of one another. They applied the SFM to estimate contacts in a working place. In [32], they estimated the number of contacts between staff members (nurse, assistant, etc.) and patients in a hemodialysis center. While these studies succeeded in capturing contacts between individuals, they didn’t account for age or heterogeneity in key parameters of the model. Furthermore, the version of the SFM used does not account for interpersonal distances between individuals [22].

The value of this distance depends on the social and cultural environment as well as the area of activity [33, 34]. Social force models are also able to accurately capture the effect of panic on the behavior of individuals.

In this work, we will use a modified social force model that is well calibrated using data relating to the movements of individuals and their interactions. Therefore we integrate desired walking velocities and interpersonal distances [33, 34] as well as demographic data in the model. We collected data for the Moroccan population. We will use the framework to assess contact patterns in workplaces, schools, residential areas/households, shopping centers and others.

## Materials and methods

### Pedestrians movement model

**Fig 1:**
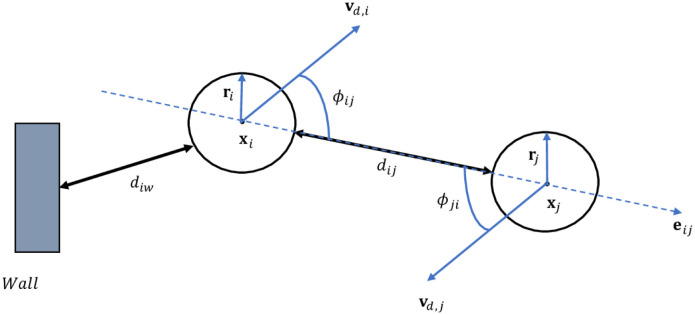
Graphical representation of pedestrian interactions. The model parameters defining the interaction between two disk-shaped pedestrians are shown in this schematic. Individuals have a center *x*_*i*_ and a radius *r*_*i*_. *d*_*ij*_ describes the distance between two individuals *i* and *j*, while *d*_*iw*_ describes the distance between an individual and a wall *w*. Each individual’s desired velocity is *v*_*d*,*i*_, and *ϕ*_*ij*_ is the angle between the direction of the desired velocity and the direction from *i* to *j*.

In the absence of contacts, regular movement of pedestrians can be described using a social force model [35]. The presented microscopic pedestrian movement model has already been introduced in details in previous works [36–38]. We use a framework that bring some modifications to the traditional expressions of repulsive forces in [19]. It introduces some parameters that make the model consistent, from a psychological point of view, with effects such as interpersonal stress (while moving, pedestrians tend to keep a certain distance between them). We represent each individual using a disk of center *x*_*i*_ and radius *r*_*i*_. The used notations for model description are graphically represented in Fig 1. We describe the motion of the *i*-th individual using the following equation:

$$\begin{array}{c} m_{i}\frac{d{\text{v}}_{i}}{dt}={\text{f}}_{i}^{self}+{\text{f}}_{i}^{soc}+{\text{f}}_{i}^{obs}, \end{array}$$ (1)

where *m*_*i*_ is the mass of the individual, **v**_*i*_ is their velocity, ${\text{f}}_{i}^{self}$ the self-driven force, ${\text{f}}_{i}^{soc}$ is a social psychological force exerted by all the other pedestrians on the individual *i*, while ${\text{f}}_{i}^{obs}$ describes his interactions with the environment. In addition to these parameters, each pedestrian has a desired velocity:

$$\begin{array}{c} {\text{v}}_{d,i}=v_{d,i}\;{\text{e}}_{d,i}, \end{array}$$ (2)

where *v*_*d*,*i*_ is its norm, and **e**_*d*,*i*_ represents the desired direction. The self-driven force allows the pedestrian to adapt his speed to the desired velocity *v*_*d*,*i*_. This force is given by the following:

$$\begin{array}{c} {\text{f}}_{i}^{self}=m_{i}\frac{{\text{v}}_{d,i}-{\text{v}}_{i}}{\tau_{i}}, \end{array}$$ (3)

*τ*_*i*_ represents the time needed for the pedestrian velocity to adjust. Helbing and Molnàr [19] introduced the original form of the social psychological force. Then, in more recent research, it was adjusted to incorporate avoidance among nearby pedestrians and permit them to maintain the desired distance [35, 39]:

$$\begin{array}{c} {\text{f}}_{i}^{soc}=\sum {\text{f}}_{ij}^{soc}, \end{array}$$ (4)

where ${\text{f}}_{ij}^{soc}$ is the social psychological force between the *i*-th and *j*-th individuals, given as follows:

$$\begin{array}{c} {\text{f}}_{ij}^{soc}=\{\begin{array}{cc} A_{soc}\;\text{exp}\;(\frac{d_{ij}-d_{soc}}{\beta soc})(\gamma +\left(1-\gamma \right)\frac{1+\text{cos}\;\phi_{ij}}{2}){\text{e}}_{ij}, & ifd_{ij}<d_{soc}\\ 0, & elsewhere \end{array}, \end{array}$$ (5)

Here, *A*_*soc*_ denotes the strength of the social psychological force, *d*_*ij*_ denotes the distance between the centers of pedestrians *i* and *j*, *d*_*soc*_ represents the desired interpersonal distance, which describes the space people prefer to maintain between them, *β*_*soc*_ represents the strength of the social psychological force’s falloff, *γ* describes the impact of interactions occurring behind the person, taken between 0 and 1, and *ϕ*_*ij*_ represents the angle between the desired velocity and **e**_*ij*_. The force ${\text{f}}_{i}^{obs}$ is the sum of the forces ${\text{f}}_{iw}^{obs}$ exerted by each wall on the individual *i*. The later is an exponential repulsive force that rises as the individual gets closer to walls:

$$\begin{array}{c} {\text{f}}_{iw}^{obs}=\{\begin{array}{cc} A_{obs}\;\text{exp}\;(\frac{d_{iw}-d_{obs}}{\beta obs}), & ifd_{iw}<d_{obs}\\ 0, & elsewhere \end{array}, \end{array}$$ (6)

Some parameters depend on the age and activity area. Their values are provided in the **data in** S2 Text. More details regarding the derivation of the social force model are provided in previous studies [19, 20, 35, 39].

### Population movement characterization data

The data presented in this work was collected during our previous work [40]. Denizens of each social context have characteristics that we consider common to the whole population and others that vary in the community. These parameters can also vary from one social context to another Table 1.

**Table 1:** Model parameters. An overview of the model parameters and their relationships with age and social setting. Each parameter’s source is also provided.

Parameter	Activity area-dependent	Age-dependent	Reference
*d*_*soc*_	yes	yes	adapted to the demographic structure [33]
*d*_*obs*_	no	no	[35, 39]
*A*_*soc*_	yes	yes	[39, 54–57]
*A*_*obs*_	no	no	[39, 54–57]
*β*_*obs*_	no	no	[39, 54–57]
*β*_*soc*_	no	no	[39, 54–57]
*v*_*d*,*i*_	yes	yes	adapted to the demographic structure [42, 43]
*m*_*i*_	yes	no	Moroccan population weight [44–47]
*γ*	no	no	[39]
*τ*_*i*_	no	no	[58, 59]

We sort the population into 16 age groups of 5 years, representing categories ranging from ‘0–5’ to ‘over 75’. The desired interpersonal distances depend on age, location, and culture. Age-specific interpersonal distances have been calculated in the literature [33, 34]. We consider three types of interpersonal distances: intimate, personal, and social distances [33]. Intimate distances are estimated between 0 and 46 cm, personal distance represents interactions where people keep a distance of 46–122 cm, and social distance describes distances from 122 to 210 cm. In our simulations, individuals maintain intimate distances in residential areas and households, personal distances in schools and workplaces, and social distances in shopping centers. Changes in the three distances caused by age were estimated in a previous study.

Here, we sample interpersonal distances from a Gaussian distribution. We consider that the desired walking velocity is heterogeneous with respect to age. We assume that it also depends on each individual’s gender, as well as the area where they walk. Some research studies provide estimates of this variable for educational, commercial, mixed, recreational, residential, and shopping areas [41, 42]. Residential and shopping areas had the lowest average walking speeds, while educational places had the fastest walking speeds [41]. Another study has shown that walking speeds are highest in workplaces and shopping centers [42]. In our work, we use the estimates provided by [42] for all locations, except in residential areas where we use the ones provided by [43].

To simulate people’s movement we used calibrated values of the social force model parameters such as the amplitude, fall off length of social forces and relaxation time. Many techniques exist in the literature to tune these parameters which we consider unrelated to age in this work Table 1. More details about these parameters and their value are available in S1 Text.

We sample the weights of the individuals from the Moroccan demographic structure [44–47]. For location-specific simulations, we sample the ages from the distributions corresponding to demographics in the corresponding location [48–53]. We consider the same density for all locations. Values of the parameters are summarized in S2 Text.

### Simulation settings

**Fig 2:**
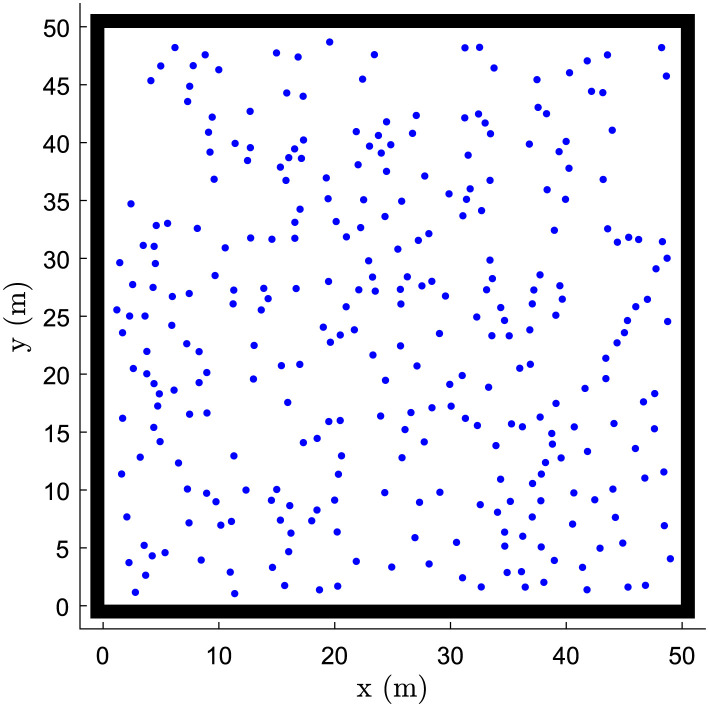
Computational domain. We didn’t add any obstacle to the domain to avoid limiting the study to a specific setting. Therefore, the places of activity have the same size.

We consider a computational domain corresponding to a space of 50 m × 50 m Fig 2. At the beginning of each simulation, 300 pedestrians are generated and randomly placed in the domain with an initial velocity set to zero. Their movement direction is random. Every 10 seconds, 30% of pedestrians alter their route. We also consider that individuals change the direction of their movement when they reach a boundary of the domain. To define a contact between two pedestrians, we consider the safety distance for respiratory infectious diseases. We then ponder the exposure time needed to inhale an infectious dose within that safety perimeter. Two pedestrians make contact when their distance stays less than 1.8 m during 30 seconds [60, 61]. When two people make first contact, any subsequent contact between the two is not counted /citeIozzi. The output of our simulations is a matrix *C*_*ij*_ that counts the total number of contacts between each age group. Then we calculate a matrix *m*_*ij*_ which gives estimates of the average number of contacts between an individual of the age group *i* and individuals of the age group *j* such that *C*_*ij*_ = *m*_*ij*_ × *n*_*i*_ = *m*_*ji*_ × *n*_*j*_ = *C*_*ji*_, where *n*_*i*_ and *n*_*j*_ are respectively the sizes of the age groups *i* and *j*. We compute the contact matrices over the duration of the activity in each of the places of activity. We consider 8 hours of activity at schools [62, 63], 5 hours in households [64], 11 hours in shopping centers [65], finally 8 hours and 16 hours in workplaces and others respectively [66].

## Results

### Sensitivity analysis

#### Sensitivity of the model to the parameter representing the walking velocity

**Fig 3:**
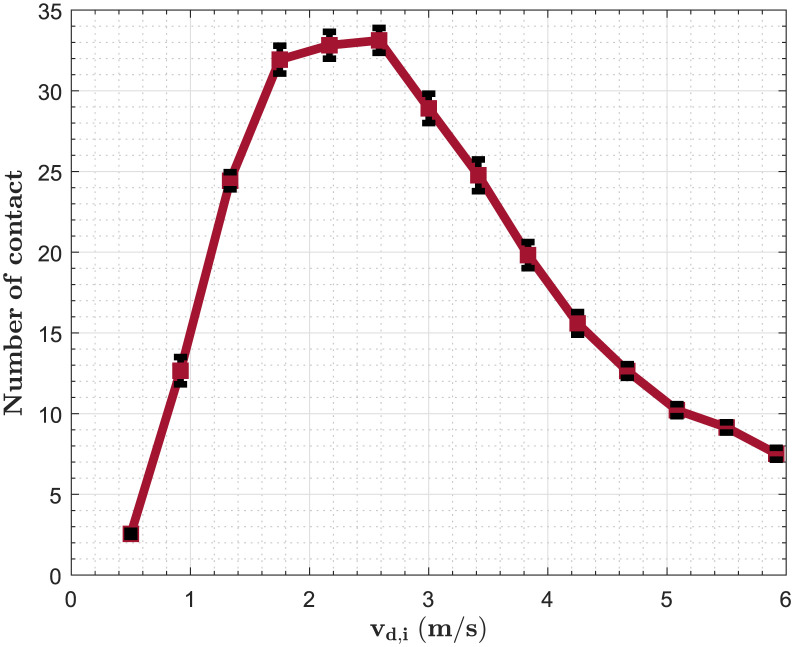
Impact of the velocity when *d*_*soc*_ = 1.22 *m*. We set the desired distance for all individuals to 1.22 m [34] and vary the velocity from 0.5 m/s to 5.92 m/s, and the amplitude of the repulsive force is *A*_*soc*_ = 700*N*.

**Fig 4:**
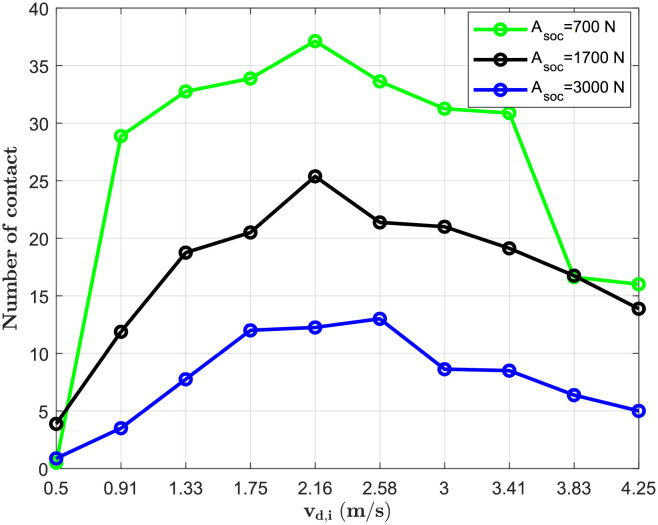
Impact of the velocity when *d*_*soc*_ = 0.2 *m*. We set the desired distance for all individuals to 0.2 m and vary the velocity from 0.5 m/s to 4.25 m/s.

To investigate the effect of velocity on the number of contacts, We set the desired distance for all individuals to 1.22 m [34] and vary the velocity from 0.5 m/s to 5.92 m/s, and the amplitude of the repulsive force is *A*_*soc*_ = 700 × *N*. Results show an increase in the average number of contact when we increase the velocity up to 2.58 m/s, and it decreases when the velocity is superior to this value Fig 3. This result could be explained by the time period during which two persons must have their distances inferior to 1.8 m in order to consider that interaction as a contact. This time in our study is 30 seconds [60]. Then, We modify the desired distance to 0.2 m Fig 4. We study the relationship between the number of contacts and the velocity for different values of the amplitude of the repulsive force *A*_*soc*_ = 700 *N*; 1700 *N*; 3000 *N*. The results show that an increase in *A*_*soc*_ flattens curbe of the number of contacts. The threshold velocity where the number of contacts start to decrease changes. It increases from 2.16 m/s for the first two values of *A*_*soc*_ to 2.58 m/s for the last value.

#### Sensitivity of the model to the parameter representing the weight

**Fig 5:**
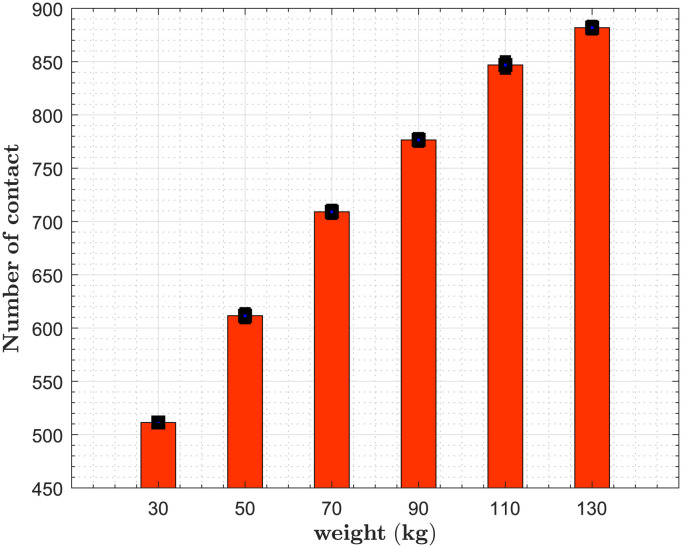
Impact of the weight. We set the desired distance for all individuals to 1.22 m and vary the weight from 30 kg to 130 kg.

We continue our investigation with the assessment of the weight. We set the desired distance for all individuals to 1.22 m [34], the desired velocity is 1.34 m/s [19, 67, 68] and the amplitude of the repulsive force is *A*_*soc*_ = 700 *N*. Results show an increase in the average number of contacts when we increase the weight Fig 5. This is due to a reduction of the social forces between pedestrians.

### Age-related contact patterns

**Fig 6:**
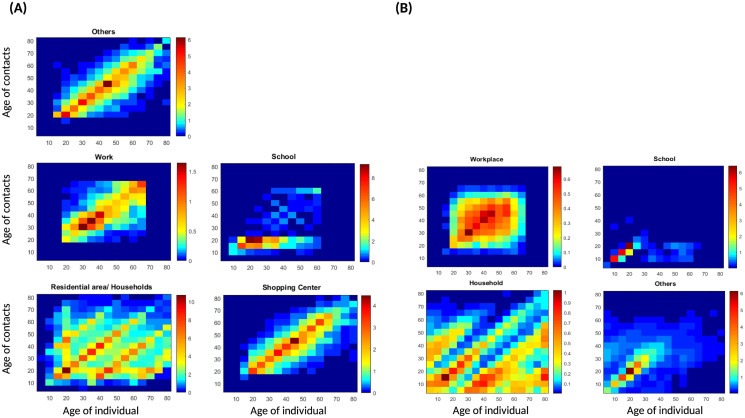
Estimate of contact patterns in the considered places of activity. Simulations data are presented in S1 and S2 Text. (**A**) contact matrices in this study. (**B**) contact matrices in [8, 9].

We estimated contact matrices with **data in** S2 Text. Fig 6A) show the contact patterns estimated in this study. Contact patterns differ from one social context to another, as do the age groups with the highest numbers of contacts. A specific age group has a high number of contacts due to small desired distances and their desired speeds Fig 3. Another parameter that can impact the number of contacts is weight Fig 5.

### Comparison with other studies

**Fig 7:**
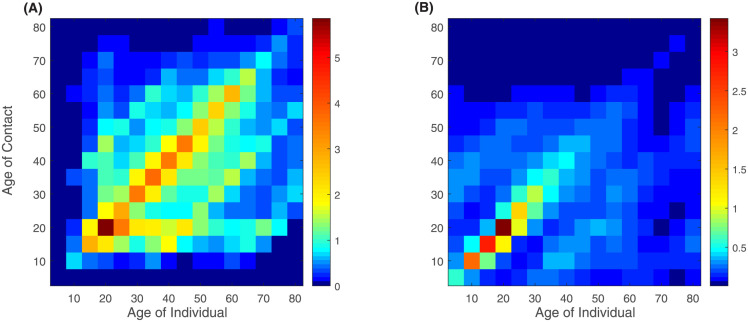
Average contact patterns. We took the average over the places of activity. (**A**) Average contact matrix in this study. (**B**) Average contact matrix in [8, 9]. The computed matrices present some similarities, in particular second diagonals over and under the main one representing contacts between parents and children.

We compare our matrices to the projected POLYMOD matrices [6, 8, 9]. Fig 6A) shows our matrices, Fig 6B) shows the estimated matrices in [8, 9] in different places and Fig 7 shows the average contact matrices.

Contacts between children and parents are clearly visible in our matrices Fig 7, A). Another pattern that appears in our matrices is contacts between the age group 25–60 and the age group 5–20, which represent interactions between students and teachers at school Fig 6. These interactions are more pronounced in our matrices compared to POLYMOD projected matrices, especially between the age groups 25–35 and 5–20. They decrease as the age of the individual increases. Our school matrix also captures interactions between teachers. The high numbers of contacts in residential areas in this study could be explained by the small desired distances (intimate distances) [33] computed in this place and the time period necessary to count an interaction as a contact (30 seconds) [60].

In [7], They measured assortativeness by the *Q* index which ranges between 0 indicating proportionate mixing and 1 indicating full mixing. *Q* = (Tr(*P*) − 1)/(*n* − 1) where *P* = [*p*_*ij*_] is the matrix whose elements *p*_*ij*_ represent the fractions of total contacts of age group *i* with age group *j* and *p*_*ij*_ = *K*_*ij*_/∑_*j*_ *K*_*ij*_. Tr(.) denotes the trace of the matrix and *N* is its size. The diagonal of *P* which is *p*_*ii*_ represents the proportion of contacts that individuals in each age group have with other individuals in the same age group. This proportion is higher in the age group 5–25 and lower in the age group 55–80 in both studies. Finally, the assortativeness index is *Q* = 0.153 in this study, and *Q* = 0.144 in [8, 9].

### Estimate of contact patterns when the population comply with social distancing

**Fig 8:**
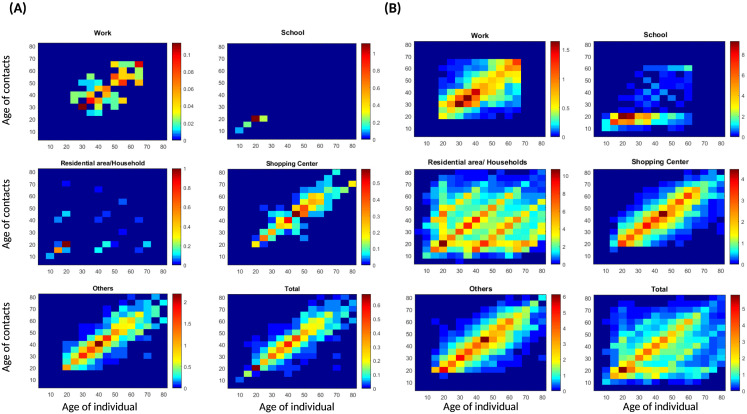
Comparison of contact patterns in social distancing settings and in absence of social distancing. **(A)** approximation of contact patterns when pedestrians complies with social distancing. The distance between denizens is 2*m* in each place. **(B)** contact patterns in absence of social distancing.

To study the effect of compliance with social distancing, we consider a homogeneous distance of 2 m between pedestrians in each social context. The age group that recorded the most contact with peers of the same category is 25–30 in the workplace, 15–20 in schools and households, 40–45 in shopping centers, and 35–40 in other settings. Overall the age groups 15–20 recorded the highest numbers of contact with people of their category Fig 8. To calculate the reduction in the number of contacts due to social distance, we use the following formula:

$$\begin{array}{c} Decrease=1-\frac{\text{average}\;\;\;\text{number}\;\;\;\text{of}\;\;\text{contact}\;\;\text{when}\;\;\text{social}\;\;\text{distance}\;\;\text{is}\;\;\text{applied}}{\text{average}\;\;\text{number}\;\;\text{of}\;\;\text{contact}\;\;\text{in}\;\;\text{a}\;\;\text{normal}\;\;\text{situation}}. \end{array}$$ (7)

**Fig 9:**
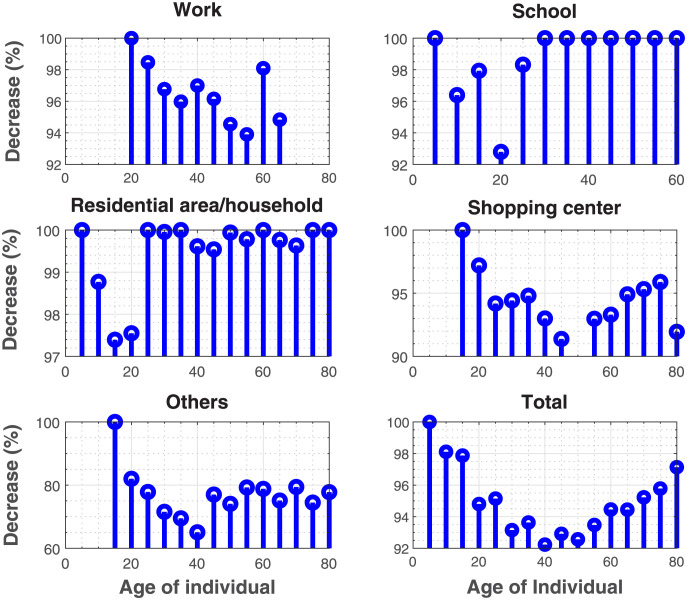
Reduction in the number of contacts of each age group due to social distancing. We estimated an average reduction in the numbers of contact of 96.6% in workplaces, 98.8% in schools, 99.5 in households, 94% in shopping centers, and 77% in public spaces/others.

Results in Fig 9 show the reduction in the numbers of contact of the different age groups, We estimated an average decrease of 96.6% in workplaces, 98.8% in schools, 99.5 in households, 94% in shopping centers, and 77% in public spaces/others. Overall, social distancing resulted in an average reduction of 95% in the number of contacts.

### Estimate of contact patterns in panic situations

**Fig 10:**
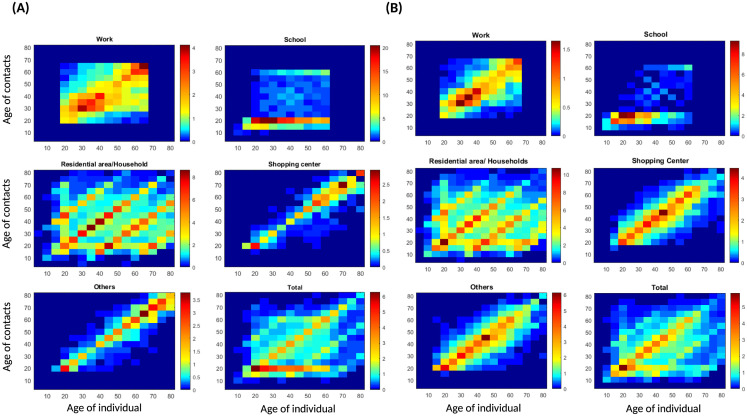
Comparison of contact patterns in a panic situation to matrices in a normal situation. **(A)** alterations of contact patterns in panic situations. In each place, pedestrians walk with a desired velocity *v*_*d*,*i*_ = 2.5 *m*/*s*. **(B)** contact patterns in a normal situation. Panic situations would increase contacts in workplaces and schools, whereas they would decrease in residential areas, households and shopping centers.

**Fig 11:**
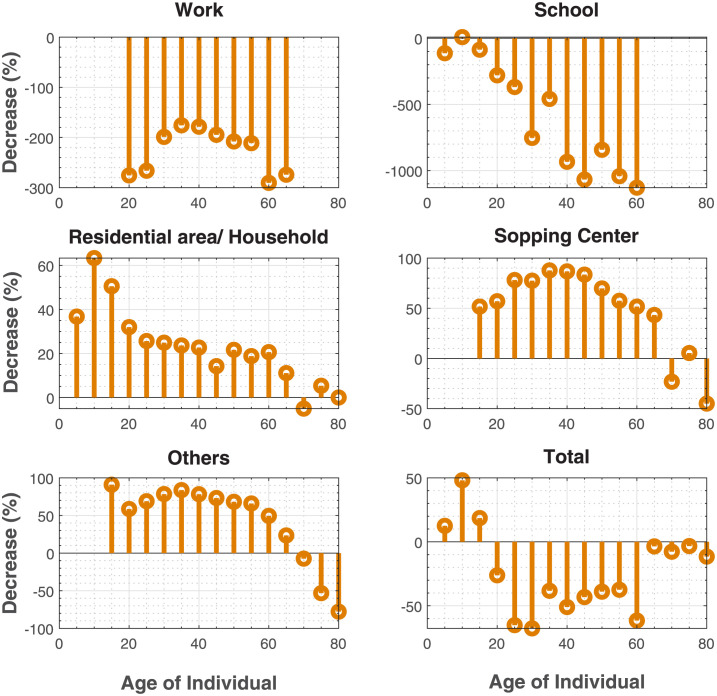
Reduction in the number of contact of the considered age groups in a panic situation. We estimated an increase of 68% in workplaces, an increase of 72% in schools, a decrease of 22% in residential areas/households, a decrease of 48% in shopping centers, and 42% in public spaces/others.

Panic situations are manifested by an increase in walking velocity and a reduction in the value of the amplitude of the repulsive forces between pedestrians. We consider a homogeneous walking desired velocity *v*_*d*,*i*_ = 2.5*m*/*s*. An individual in the age group 60–65 will have the highest number of contacts with individuals in the same age group at work, an individual in the age group 20–25 will have the highest number of contacts with individuals in the age group 15–20 at school, an individual in the age group 30–35 and his fellows in residential areas/households, an individual aged 65–70 and his fellows in shopping centers, and last of all, an individual in the age group 60–65 and his peers in other places. Conclusively, considering all the places of activity the results indicate that an individual in the age group 15–20 would record the highest number of contacts with individuals of the same age group Fig 10. According to Fig 11, panic situations would increase contacts in workplaces and schools, whereas they would decrease in residential areas, households and shopping centers. Results suggest a decrease of -227% (an increase of 68%) in workplaces, a decrease of -589% (an increase of 72%) in schools, a decrease of 22% in residential areas/households, 48% in shopping centers, and 42% in public spaces/others. Overall, we observe a decrease of -23% (an increase of 11%) in all places. This result could be explained by differences in the desired distances in the places of activity. In our simulations we considered personal distances estimated in [33], whilst we considered smaller distances (intimate distances) in residential areas/households [33], and longer distances (social distances) in shopping centers and others [33]. Consequently, our matrix in a panic situation is dominated by school contact.

## Discussion

The calculation of contacts between individuals is central to the predictions made by epidemiological models. We present a novel method that uses available data related to movements and interactions between individuals. Our method is based on a well-calibrated social force model [35, 39, 54, 57]. The framework enables the integration of data on interpersonal distances, walking speed and demographics. This approach allows us to define contact from a safe distance and the time that is necessary to inoculate an infectious dose of virus. Recent advances in estimating contact patterns represent the POLYMOD project and Little Italy. These two methods present two different definitions of contact. One takes into account physical contact as well as conversations longer than three words. The other defines contact as sharing the same space. Prior studies that estimated contact patterns for infectious respiratory diseases used the first definition of contact. Our matrices are calculated over a definition that accounts for a safety distance and an exposure time that is needed to inoculate an infectious dose of a virus. Although these parameters are not available for most infectious respiratory diseases, they have been estimated for COVID-19 [60, 61].

The estimated matrices in a normal situation in this study are highly assortative and share some similarities with the POLYMOD matrices projected on Morocco [6, 8]. Indeed, the contacts between children and parents are clearly visible in our matrices. The contacts of children and adolescents are more assortative when compared with other age groups.

Our results suggest heterogeneity in contact patterns with respect to age when social distancing is imposed. This accord with the idea that social distancing create social heterogeneity, and explain why it seems that this measure is not enough to control the COVID-19 epidemic [15]. Studies based on surveys present less variability in contact patterns when social distancing is imposed [16–18]. We have also been able to identify the age groups whose numbers of contacts will remain substantial in each social context. The age group 25–30 recorded the highest number of contacts with peers of the same category in the workplace, 15–20 in schools and households, 40–45 in shopping centers, and 35–40 in other settings.

In our studies of panic situations, we found that its effects on contact patterns varied depending on the social context. Findings suggest that panic situations would increase contacts in workplaces of 68% and 72% in schools, whereas they would decrease in residential areas of 22%, 48% in shopping centers, and 42% in public spaces/others. Overall, we estimated an increase of 11% considering all places. This variability in the different places of activity could be explained by differences in desired distances. An average increase in the number of contacts was only observed in places where personal distances were computed (workplace and school) [33]. In Residential areas where smaller distances (intimate distances) are computed, we observed a reduction in the number of contacts as well as shopping centers and Others were longer distances (social distance) are computed [33]. We chose a velocity of 2.5 m/s in our simulations of panic. This velocity is a threshold where the number of contacts starts to decrease Figs 3 and 4. When interpersonal distances are also high (social distances), contacts don’t last long. So, the model cannot capture them since the time to consider a contact is 30 seconds. As shown in the sensitivity analysis, when the distances are too small (intimate distances), the threshold velocity is inferior to or nearly equal to 2.5 m/s Fig 4. This explains the decrease in the number of contacts in households. Although the local density (number of person per *m*^2^) is variable throughout a simulation, increasing the global density could change the variability observed due to the time of exposure computed in this study (30 seconds).

It is important to note that the model was parameterized to describe the movement of Moroccans in different areas of activity. This was done in order to make the conclusions useful for local public health officials. However, we had difficulty finding all data on the movement of the Moroccan population and we had to use values corresponding to other countries in some cases. However, this method could be an alternative to direct observations and could help estimate children’s contact patterns which are typically assessed through parental proxy reporting in surveys [6].

## Conclusion

We provided a definition of contact that is realistic, involving interpersonal distances and the duration of proximity between individuals. The simulations can be adapted to estimate contact patterns and whether one or the other of these parameters is important for the considered infection. The interpersonal distance and duration of proximity chosen in the definition can be directly calibrated using serological data to capture infection dynamics. Such versatility is difficult with other definitions.

Comparisons between different studies are usually based on features of the estimated contact matrices, which directly impact infection dynamics. These particularities are the number of contacts, the presence of secondary diagonals, the presence of contacts between students and teachers at school, and the assortativity estimated using an index. An analysis of these features reveals a good agreement with the POLYMOD projected matrices. In this study, we did not make a comparison with Little Italy since their contact patterns are estimated for Italy. However, the latter also allowed for the estimation of highly assortative matrices.

Our results suggest variability in population responses when social distancing is imposed. We used demographic and socio-cultural data, which are integrated into the model, to simulate interactions between individuals. Social distancing in the model is taken into account by forcing an interpersonal distance of 2 m between people. Other model parameters remain unchanged. The observed heterogeneity could be explained by the demography, the velocity, and the amplitudes of the social-psychological forces. Several studies have tried to identify factors leading to this heterogeneity in social distancing [69–71]. Socioeconomic status is sometimes an issue, communities below the poverty level as well as essential workers have difficulty adhering to the measures [69]. In [70], they suggest that demographic factors such as gender and age determine the level of adherence to social distancing. In our study, age also seems to be a determining factor, especially in schools where the level of adherence is much higher among older individuals. The disparity observed in the responses to social distancing could explain its relative ineffectiveness [15]. Indeed, in [72] the researchers showed that the relationship between the heterogeneity in social distancing and the recovery pace is significant. The reduction in heterogeneity would reduce the number of cases and delay the peak of the COVID-19 pandemic. Some studies indicate that the compliance of only certain groups of the population is ineffective and almost as bad as when no preventative measures are taken [73, 74]. Although social distancing is not as effective, investigations estimate that without its introduction, in the long term, COVID-19 would have increased exponentially across regions in China [75]. They recommend a combination of non-pharmaceutical interventions to contain the disease. Another evidence promote the combination of multiple intervention strategies [76]. This analysis supposes that the combinations of NPIs present greater effectiveness only if they include social distancing.

Although the interpersonal distances and walking velocities were not specifically estimated for Morocco, they are based on data from a meta-analysis. We used data in [33] for interpersonal distances and [42] for velocities. The study [33] included two African countries in its analysis. In [42], they included two Arabic countries, with one being another north African country. We believe the results presented are reliable and relevant for local public health decisions. This work provides an interesting method for the estimation of social contact matrices, which can be ameliorated in future research to integrate newly estimated data.

In future works designed to forecast different epidemiological scenarios, we will integrate the estimated matrices into a compartmental model to simulate infection dynamics. The obtained results will be compared with studies using other approaches to the integration of social distancing.

## Supporting information

S1 Text Details on the social force model parameters. (PDF)

S2 Text Data on model parameters for each social context. (PDF)
