# Supplementary material for: Morocco’s population contact matrices: A crowd dynamics-based approach using aggregated literature data
Source: PLoS One. 2024 Mar 14;19(3):e0296740. doi: 10.1371/journal.pone.0296740 (PMC10939283; doi:10.1371/journal.pone.0296740)
Supplement: S1 Text — (PDF) [file pone.0296740.s001.pdf]

Supplementary Text 1

Morocco's population contact matrices: A crowd dynamics-based approach using aggregated literature data

DRAMANE SAM IDRIS KANTÉ [1,2], AISSAM JEBRANE [1], ADNANE BOUKAMEL [1], ABDELILAH HAKIM [2]

[1] Complex Systems and Interactions Team, Ecole Centrale Casablanca, Ville Verte, Bouskoura 27182, Morocco
[2] LAMAI, Faculty of Sciences and Technology, Cadi Ayyad University, Marrakesh 40140, Morocco
*Corresponding author: dramane.kante@centrale-casablanca.ma

1. CALIBRATION OF THE SOCIAL FORCE MODEL IN NORMAL CONDITIONS

Several methods were used to identify the values of physical parameters involved in social force models [1–3]. For example, some previous works [1, 2] used fundamental diagram to identify these parameters. This diagram assumes a relation between the average walking speed and the density of the crowd. Analytical methods were also used for the determination of parameters. In these methods, parameters were fitted such that the movement characteristics of agents approximate those of pedestrians in organized pedestrian experiments [3] or in real situations [4]. The social force model was calibrated to reproduce pedestrian behaviors in normal situations. [5] estimated model parameters to analyze displacements in urban settings. [6] tuned the parameters to simulate scattered crowds in normal situations. [7] modified the model to simulate pedestrian twice crossing behavior. [8] used the model to study pedestrian counterflow. Panic situations also have been extensively studied [6, 9–11]. In panic situations, we use the collision model described in [12, 13] to avoid collision between pedestrians. Panic situations are described through an elevation in the mean desired velocity and the tuning of other parameters, as reported in previous studies [14, 15]. We compute the values of τ_i, A_{obs}, β_{obs}, and γ regardless of the scenario (normal, social distancing, or panic).

Table S1. Numerical values of some parameters regardless of the scenario

Parameter	Value	Reference
τ_i	0.5 (s)	[6, 16]
A_{obs}	1000 (N)	[9, 12, 13]
β_{obs}	0.8 (m)	[9, 12, 13]
γ	$]0,1[$	[6, 12, 13]

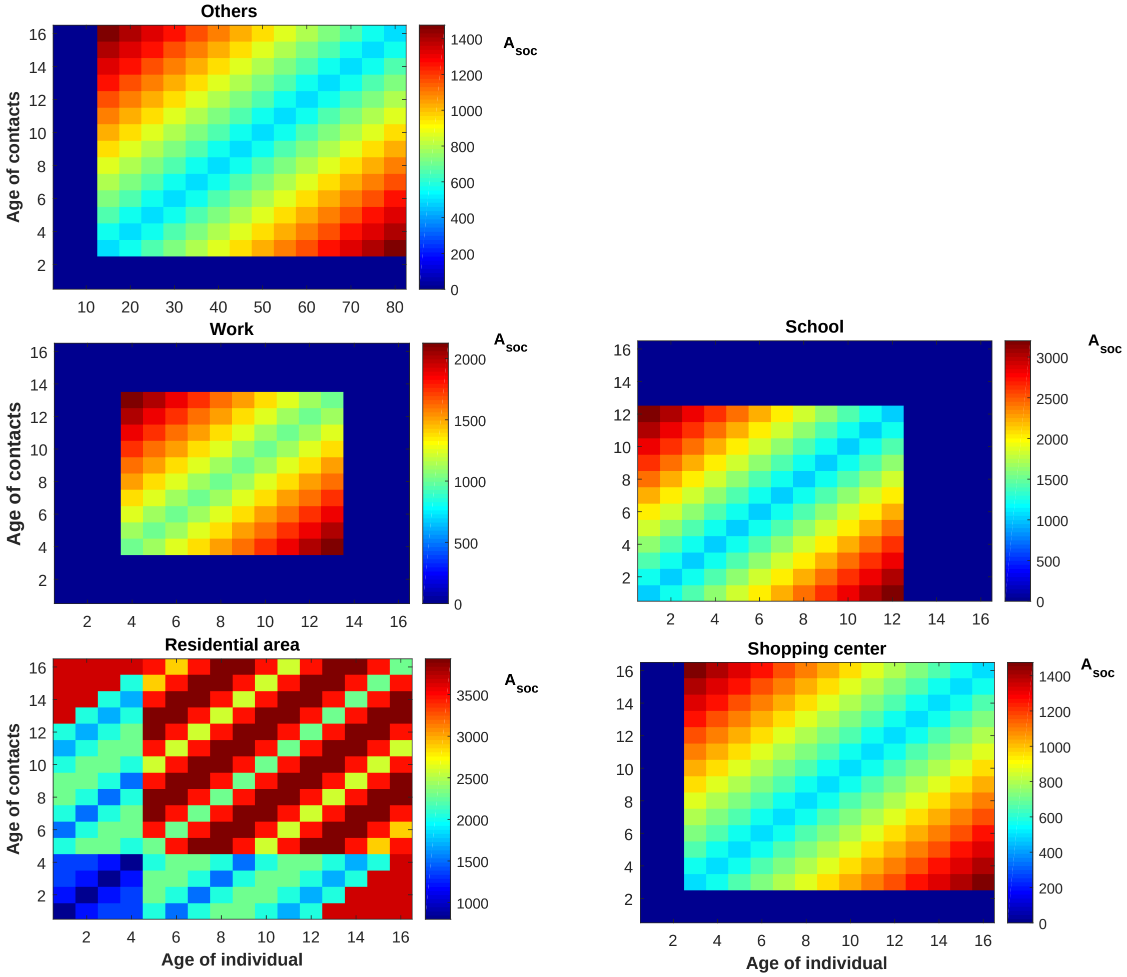

Fig. S1. Magnitude of the social force between age groups in normal and social distancing scenarios. The values corresponding to zero in the matrices of social force magnitude in Others, Work, School, and Shopping center represent age groups that do not visit these places.

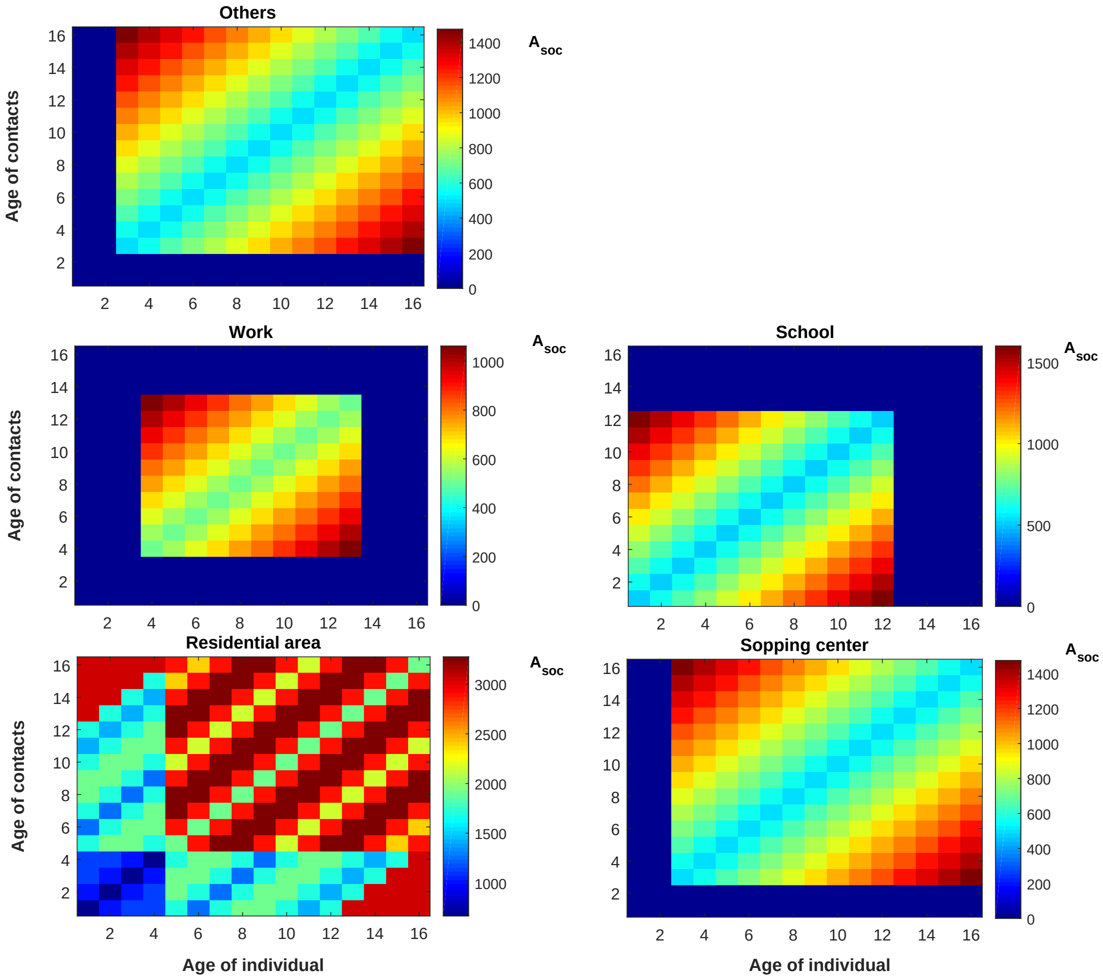

Fig. S2. Magnitude of the social force between age groups in panic situations. The values corresponding to zero in the matrices of social force magnitude in Others, Work, School, and Shopping center represent age groups that do not visit these places.

REFERENCES

1. E. Bassoli and L. Vincenzi, "Parameter calibration of a social force model for the crowd-induced vibrations of footbridges," Front. Built Environ. **7**, 75 (2021).
2. N. Taherifar, H. Hamedmoghadam, S. Sree, and M. Saberi, "A macroscopic approach for calibration and validation of a modified social force model for bidirectional pedestrian streams," Transp. A: Transp. Sci. **15**, 1637–1661 (2018).
3. T. Kretz, J. Lohmiller, and P. Sukennik, "Some indications on how to calibrate the social force model of pedestrian dynamics," Transp. Res. Rec. **2672**, 228–238 (2018).
4. S. Seer, C. Rudloff, T. Matyus, and N. Brändle, "Validating social force based models with comprehensive real world motion data," Transp. Res. Procedia **2**, 724–732 (2014).
5. A. Johansson, D. Helbing, and P. K. Shukla, "Specification of the social force pedestrian model by evolutionary adjustment to video tracking data," Adv. complex systems **10**, 271–288 (2007).
6. P. Wang, "Understanding social-force model in psychological principles of collective behavior," arXiv preprint arXiv:1605.05146 (2016).
7. Y. Guo, S. Ma, F. Wei, L. Lu, F. Sun, and J. Wang, "Analysis of behavior characteristics for pedestrian twice-crossing at signalized intersections based on an improved social force model," Sustainability **14**, 2003 (2022).

8. S. Heliövaara, T. Korhonen, S. Hostikka, and H. Ehtamo, "Counterflow model for agent-based simulation of crowd dynamics," Build. Environ. **48**, 89–100 (2012).
9. I. Sticco, G. Frank, and C. Dorso, "Social force model parameter testing and optimization using a high stress real-life situation," Phys. A: Stat. Mech. its Appl. **561**, 125299 (2021).
10. R. Zhou, Y. Cui, Y. Wang, and J. Jiang, "A modified social force model with different categories of pedestrians for subway station evacuation," Tunn. Undergr. Space Technol. **110**, 103837 (2021).
11. Z. Yuan, H. Jia, L. Zhang, and L. Bian, "A social force evacuation model considering the effect of emergency signs," SIMULATION **94**, 723–737 (2018).
12. B. Kabalan, P. Argoul, A. Jebrane, G. Cumunel, and S. Erlicher, "A crowd movement model for pedestrian flow through bottlenecks," Annals Solid Struct. Mech. **8**, 1–15 (2016).
13. A. Jebrane, P. Argoul, A. Hakim, and M. E. Rhabi, "Estimating contact forces and pressure in a dense crowd: Microscopic and macroscopic models," Appl. Math. Model. **74**, 409–421 (2019).
14. D. Helbing, I. Farkas, and T. Vicsek, "Simulating dynamical features of escape panic," Nature **407**, 487–490 (2000).
15. D. Helbing, I. Farkas, P. Molnar, and T. Vicsek, *Simulation of pedestrian crowds in normal and evacuation situations* (2002), vol. 21, pp. 21–58.
16. X. Yang and Q. Wang, "Crowd hybrid model for pedestrian dynamic prediction in a corridor," IEEE Access **7**, 95264–95273 (2019).
