# Supplementary material for: Morocco’s population contact matrices: A crowd dynamics-based approach using aggregated literature data
Source: PLoS One. 2024 Mar 14;19(3):e0296740. doi: 10.1371/journal.pone.0296740 (PMC10939283; doi:10.1371/journal.pone.0296740)
Supplement: S2 Text — (PDF) [file pone.0296740.s002.pdf]

Supplementary Text 2

Morocco's population contact matrices: A crowd dynamics-based approach using aggregated literature data

Dramane Sam Idris Kanté [1,2], Aissam Jebrane [1], Adnane Boukamel [1], Abdelilah Hakim [2]

[1] *Complex Systems and Interactions Team, Ecole Centrale Casablanca, Ville Verte, Bouskoura 27182, Morocco*
[2] *LAMAI, Faculty of Sciences and Technology, Cadi Ayyad University, Marrakesh 40140, Morocco*
**Corresponding author: dramane.kante@centrale-casablanca.ma*

Table S2. Collected data for shopping centres.

Shopping centre			
Age bracket	Demography (%)	Desired speed (m/s)	Desired distance (m)
[0,5[	0	$N(0.88; 0.106)$	$N(1.99; 0.056)$
[5,10[	0.6	$N(1.15; 0.04)$	
[10,15[	1	$N(1.20; 0.048)$	$N(2.04; 0.075)$
[15,20[	12.75	$N(1.36; 0.036)$	
[20,25[	12.25	$N(1.42; 0.035)$	$N(2.16; 0.072)$
[25,30[	12.50		
[30,35[	12.04	$N(1.42; 0.051)$	$N(2.19; 0.0814)$
[35,40[	11.42		
[40,45[	10.03	$N(1.39; 0.065)$	$N(2.15; 0.076)$
[45,50[	6.05		
[50,55[	5.43	$N(1.30; 0.023)$	$N(2.11; 0.066)$
[55,60[	5.11		
[60,65[	4.49	$N(1.26; 0.023)$	$N(2.12; 0.073)$
[65,70[	3.44		
[70,75[	2.08	$N(1.01; 0.003)$	$N(2.24; 0.047)$
[75,80[	1.35		

Table S3. Collected data for schools.

School			
Age bracket	Demography (%)	Desired speed (m/s)	Desired distance (m)
[0,5[	0.885	$N(0.65; 0.106)$	$N(1.26; 0.125)$
[5,10[	30.97	$N(1.08; 0.028)$	
[10,15[	38.05	$N(1.33; 0.012)$	$N(1.31; 0.135)$
[15,20[	22.12	$N(1.45; 0.035)$	
[20,25[	0.885	$N(1.44; ; 0.023)$	$N(1.35; 0.141)$
[25,30[	0.885	$N(1.39; 0.246)$	
[30,35[	0.885	$N(1.36; 00.0304)$	$N(1.38; 0.108)$
[35,40[	0.885	$N(1.33; 0.030)$	
[40,45[	0.885	$N(1.3; 0.021)$	$N(1.36; 0.128)$
[45,50[	0.885	$N(1.26; 0.021)$	
[50,55[	0.885	$N(1.16; 0.243)$	$N(1.22; 0.015)$
[55,60[	1.77	$N(1.2; 0.015)$	
[60,65[	0	$N(1.2; 0.018)$	$N(1.37; 0.129)$
[65,70[	0	$N(1.18; 0.018)$	
[70,75[	0	$N(1.08; 0.015)$	$N(1.49; 0.1309)$
[75,80[	0	$N(0.92; 0.015)$	

Table S4. Collected data for residential areas/households.

Residential area/Household			
Age bracket	Demography (%)	Desired speed (m/s)	Desired distance (m)
[0,5[	11.55	$N(0.88; 0.106)$	$N(0.38; 0.095)$
[5,10[	12.23	$N(1.53; 0.447)$	
[10,15[	11.42		$N(0.41; 0.088)$
[15,20[	07.45	$N(1.55; 0.323)$	
[20,25[	07.17		$N(0.41; 0.097)$
[25,30[	07.53		
[30,35[	07.25	$N(1.47; 0.246)$	$N(0.42; 0.099)$
[35,40[	06.89		
[40,45[	06.05		$N(0.45; 0.126)$
[45,50[	05.39		
[50,55[	04.83	$N(1.38; 0.243)$	$N(0.41; 0.103)$
[55,60[	04.55		
[60,65[	03.03		$N(0.47; 0.144)$
[65,70[	02.34	$N(1.16; 0.255)$	
[70,75[	01.41		$N(0.49; 0.149)$
[75,80[	0.92		

Table S5. Collected data for workplaces.

Workplace			
Age bracket	Demography (%)	Desired speed (m/s)	Desired distance (m)
[0,5[	0	$N(0.88; 0.106)$	$N(1.26; 0.125)$
[5,10[	0	$N(1.16; 0.043)$	
[10,15[	0	$N(1.21; 0.046)$	$N(1.31; 0.135)$
[15,20[	7.75	$N(1.39; 0.035)$	
[20,25[	7.65	$N(1.35; 0.023)$	$N(1.35; 0.141)$
[25,30[	15.34		
[30,35[	14.41	$N(1.43; 0.030)$	$N(1.38; 0.108)$
[35,40[	12.20		
[40,45[	10.35	$N(1.434; 0.021)$	$N(1.36; 0.128)$
[45,50[	9.02		
[50,55[	8.38	$N(1.433; 0.015)$	$N(1.30; 0.131)$
[55,60[	07.90		
[60,65[	7.00	$N(1.339; 0.018)$	$N(1.37; 0.129)$
[65,70[	0		
[70,75[	0	$N(1.262; 0.015)$	$N(1.49; 0.130)$
[75,80[	0		

Table S6. Collected data for public spaces/other locations.

Public space/other locations			
Age bracket	Demography (%)	Desired speed (m/s)	Desired distance (m)
[0,5[	0	$N(0.88; 0.1061)$	$N(1.99; 0.056)$
[5,10[	0.6	$N(1.11; 0.028)$	
[10,15[	1	$N(1.35; 0.012)$	$N(2.04; 0.075)$
[15,20[	12.75	$N(1.39; 0.035)$	
[20,25[	12.25	$N(1.34; 0.038)$	$N(2.16; 0.072)$
[25,30[	12.50		
[30,35[	12.04	$N(1.26; 0.032)$	$N(2.19; 0.0814)$
[35,40[	11.42		
[40,45[	10.03	$N(1.26; 0.0478)$	$N(2.15; 0.076)$
[45,50[	6.05		
[50,55[	5.43	$N(1.23; 0.041)$	$N(2.11; 0.066)$
[55,60[	5.11		
[60,65[	4.49	$N(1.21; 0.049)$	$N(2.12; 0.073)$
[65,70[	3.44		
[70,75[	2.08	$N(0.95; 0.23)$	$N(2.24; 0.047)$
[75,80[	1.35		

Table S7. Weight and relaxation time of the Moroccan population.

Age bracket	Weight (kg)
[0,5[	$N(13.10; 4.05)$
[5,10[	$N(20.91; 2.935)$
[10,15[	$N(34.525; 6.553$
[15,20[	$N(50.532; 7.196$
[20,25[	$N(60.95; 10.45)$
[25,30[	$N(66.05; 13.25)$
[30,35[	$N(73.25; 14.75)$
[35,40[	
[40,45[	$N(73.55; 16.25)$
[45,50[	
[50,55[	$N(73.10; 23.00)$
[55,60[	
[60,65[	$N(72.1; 13.63)$
[65,70[	$N(71.99; 13.63)$
[70,75[	$N(67.05; 15.50)$
[75,80[	$N(70.39; 16.71)$
